# Ferritinophagy, a form of autophagic ferroptosis: New insights into cancer treatment

**DOI:** 10.3389/fphar.2022.1043344

**Published:** 2022-10-21

**Authors:** Kai Sun, Chenyuan Li, Shichong Liao, Xinrui Yao, Yang Ouyang, Yi Liu, Zhong Wang, Zhiyu Li, Feng Yao

**Affiliations:** ^1^ Department of Breast and Thyroid Surgery. Renmin Hospital of Wuhan University, Wuhan, China; ^2^ School of Science, University of Sydney, Sydney, New South Wales, NSW, Australia

**Keywords:** ferroptosis, ferritinophagy, cancer, iron, autophagy

## Abstract

Ferritinophagy, a form of autophagy, is also an important part of ferroptosis, a type of regulated cell death resulting from abnormal iron metabolism involving the production of reactive oxygen species. As ferroptosis, autophagy and cancer have been revealed, ferritinophagy has attracted increasing attention in cancer development. In this review, we discuss the latest research progress on ferroptosis, autophagy-associated ferroptosis led by ferritinophagy, the regulators of ferritinophagy and promising cancer treatments that target ferritinophagy. Ferritinophagy is at the intersection of ferroptosis and autophagy and plays a significant role in cancer development. The discussed studies provide new insights into the mechanisms of ferritinophagy and promising related treatments for cancer.

## 1 Introduction

Ferroptosis is a form of regulated cell death that, is highly related to reactive oxygen species (ROS) and lipid metabolism (Lambeth and Neish, 2014). Autophagy, a self-degradative process that is, important for cellular homeostasis, is divided into three main types: macroautophagy, microautophagy, and chaperone-mediated autophagy (Santana-Codina and Mancias, 2018). Researchers have found that ferroptosis is strongly correlated with autophagy and cancer, and previous studies have revealed ferritinophagy; this review places an emphasis on ferritinophagy (Kang and Tang, 2017). Ferritinophagy is a new autophagy process associated with ferroptosis in which the binding of nuclear receptor coactivator 4 (NCOA4) and ferritin in autophagosomes (Dixon et al., 2012) causes autophagic degradation (Tang et al., 2018). Research related to cancer and ferritinophagy conducted over the past few years has shown new intracellular processes and mechanisms connecting iron and cancer (Manz et al., 2016). An increasing number of studies have shown that ferritinophagy can influence the growth of tumors (Su et al., 2020; Chen et al., 2021). In this review, we mainly discuss the process of ferritinophagy, its regulators and probable treatments for cancers that target ferritinophagy.

## 2 Latest progress in ferroptosis

### 2.1 Ferroptosis regulates different diseases

Ferroptosis is an iron-induced, lipid peroxide-driven form of programmed cell death (Stockwell et al., 2017). Ferroptosis is distinguished from other forms of cell death by morphological features, including smaller, abnormally shaped mitochondria with decreased and flat cristae, condensed mitochondrial membranes, and a ruptured outer membrane (Gao et al., 2019). Membrane oxidative damage is the final step of ferroptosis, and triggers of this process have been identified. Lethal lipid peroxide (LPO), which is cytotoxic, is the primary cause of ferroptosis (Li et al., 2021a). Over the past decades, different diseases, including cardiovascular diseases, ischemia–reperfusion injury, acute kidney injury and neural diseases, particularly cancer, have been found to be closely associated with ferroptosis (Hambright et al., 2017; Chen et al., 2020; Lee et al., 2020; Wang et al., 2020; Shao et al., 2021; Wang et al., 2021).

### 2.2 Typical GPX4-mediated ferroptosis

**FIGURE 1:**
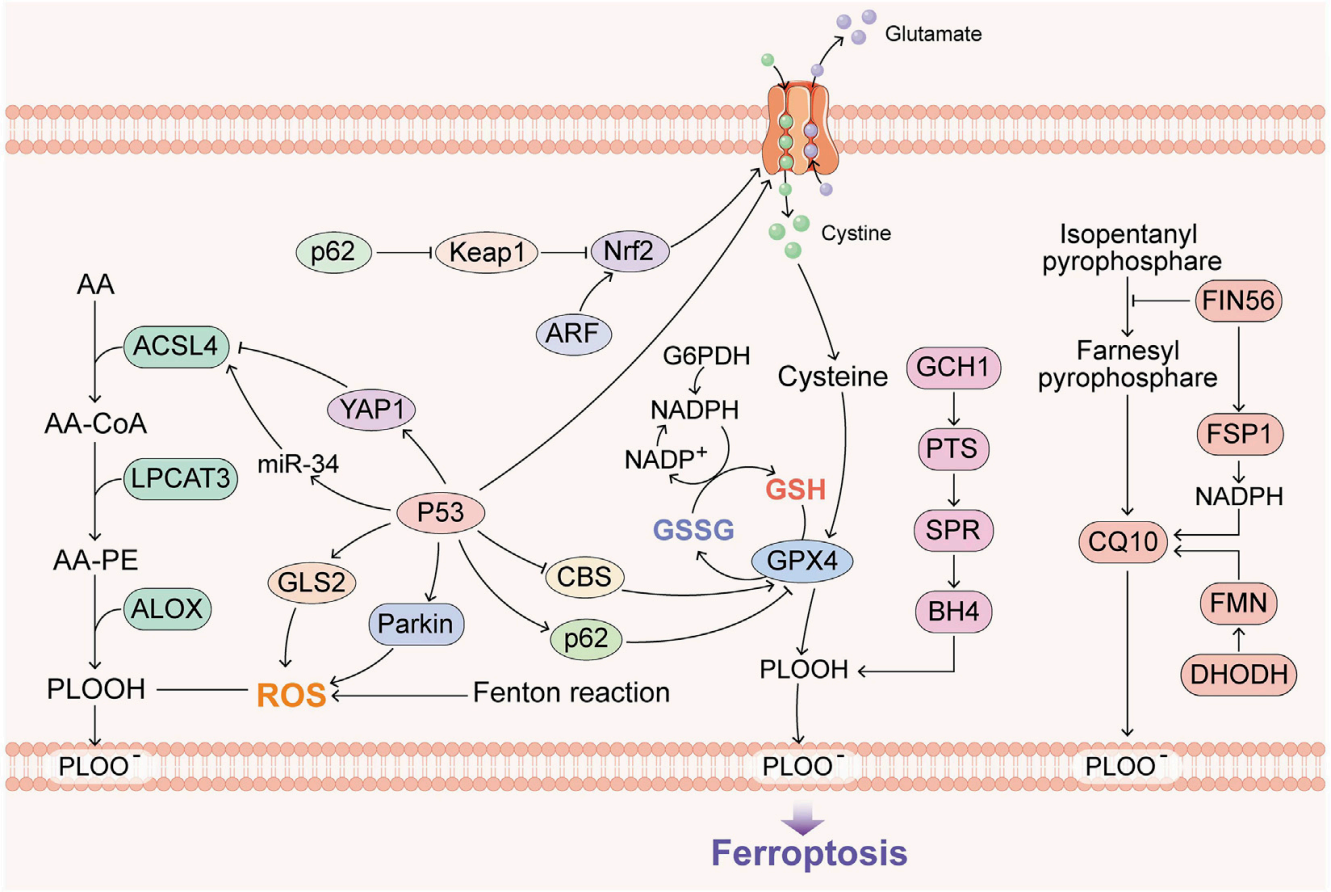
Overview of the recent research progress on ferroptosis. GPX4, BH4, FSP1 and DHODH constitute the main defense system of ferroptosis. ACSL4 and the Fenton reaction promote ferroptosis, and p53 has dual roles in ferroptosis.

Ferroptosis was first identified by the discovery of erastin and RSL3 (Dixon et al., 2012). Cell death induced by the compounds occurs in the absence of the core apoptosis machinery caspases BAX and BAK. The knockdown of RIPK1/RIPK3, CASP1/4 and PARP1 also has no influence on ferroptosis, which means that this type of cell death is distinct from apoptosis, necroptosis, pyroptosis and parthanatos (Yagoda et al., 2007; Yang and Stockwell, 2008; Friedmann Angeli et al., 2014). Further studies have demonstrated that the function of erastin in this process is to inhibit the xc-system and that RSL3 inhibits GPX4; the former is a cysteine/glutamate transporter, and the latter is known as a phospholipid hydroperoxidase (Yang et al., 2014). Recent studies have identified more ferroptotic inducer targets, such as erastin2, IKE, sorafenib, sulfasalazine, glutamate, FIN56, ML162, ML210, FINO2 and DPIs. Targeting xc-/GPX4 has had a great impact on diseases related to ferroptosis (Louandre et al., 2013; Gaschler et al., 2018; Zhang et al., 2019; Sun et al., 2021a; Koppula et al., 2021; Moosmayer et al., 2021; Zhou et al., 2021). Moreover, typical signaling pathways, such as p62-Keap1-Nrf2, have been proven to regulate the xc-system, which offers a target (Pietsch et al., 2003; Shin et al., 2018; Dodson et al., 2019; Qiang et al., 2020) (Figure 1).

### 2.3 GPX4-independent ferroptosis

Specifically, GPX4, FSP1 and BH4 are considered to constitute three major ferroptosis defense systems (Doll et al., 2019). FSP1 is a GPX4-independent ferroptosis suppressor, and FSP1 is also called apoptosis-inducing factor mitochondria-associated 2 (AIFM2), which confers protection against ferroptosis in the presence of GPX4 deletion (Shimada et al., 2016; Bersuker et al., 2019). Further research has demonstrated that its suppression is mediated by CoQ10. FSP1 can catalyze the regeneration of CoQ10 using NAD(*p*)H to ensure lipid peroxidation (Bersuker et al., 2019; Kraft et al., 2020). BH4 is also independent of the two other pathways, and its antioxidant capacity is shown by capturing free radicals and transferring them to BH2 (Soula et al., 2020). GCH1 catalyzes the rate-limiting step in the production of BH4, and GCH1-knockout cells are not sensitive to erastin treatment or other ROS-inducing compounds, which means that BH4 specifically confronts lipid peroxidation (Soula et al., 2020). Subsequently, an increasing number of discoveries on GPX4-independent ferroptosis have been revealed.

Recently, the mechanisms of p53 in inducing ferroptosis have been revealed, and both the canonical (GPX4-dependent) and noncanonical (GPX4-independent) ferroptosis pathways, as typical transcription factors, provide room for the imagination of ferroptosis and cancer (Jiang et al., 2015). DHODH was also found to operate in parallel with mitochondrial GPX4, independent of cytosolic GPX4 and FSP1, for reducing CoQ to CoQ10. The DHODH inhibitor brequinar has been applied to low-GPX4 tumors, and combined treatment with brequinar and sulfasalazine can inhibit high-GPX4 tumors (Mao et al., 2021).

### 2.4 Redox lipid death: Fenton reaction and PUFAS

Iron plays an important role in mammalian cells because it participates in cell metabolism, proliferation and growth (Ye et al., 2021). These processes are conducted by a variety of proteins that contain iron and hemoglobin, including many proteins that are important for cell growth (Federico et al., 2022). Iron in the human body exists in two oxidation states: ferrous iron and ferric iron (Smith et al., 2022). Iron can gain and lose electrons, which enables it to trigger the Fenton reaction, and during this reaction, Fe2+ donates an electron to hydrogen peroxide (H2O2) to form the hydroxyl radical (•HO), which is an ROS (Thomas et al., 2009). Elevated iron can cause the generation of ROS, which can damage lipids, proteins, RNA and DNA, and eventually leads to tumor growth (Zhou et al., 2011). Various types of cancers, such as lung cancer, breast cancer, prostate cancer, pancreatic cancer, colorectal cancer, hepatocellular cancer, renal cell carcinoma, and melanoma, are promoted by iron (Yan et al., 2021a). Additionally, iron can promote DNA replication and repair (Zhou et al., 2018). In addition, in 2016, ACSL4, which can enrich cellular membranes with PUFAs and alter the cellular lipid composition, was found to sensitize breast cancer cells to ferroptosis during RSL3 treatment (Doll et al., 2017). Thus, decreasing the cellular iron levels and targeting iron metabolic pathways and lipid metabolism may provide new tools for cancer therapy (Bordini et al., 2020).

## 3 Different types of ferroptosis evasion processes promote cancer development

### 3.1 The typical GPX4 pathway regulates cancer development

Ferroptosis is regulated by oncogenes, and the most frequently mutated gene, p53, as noted above, has been proven to regulate ferroptosis in dual manners (Jiang et al., 2015; Xie et al., 2017). In addition, BAP1, KEAP1, KRAS and ARF can downregulate SLC7A11 through NRF2 or NRF2-independent pathways (Chen et al., 2017; Dodson et al., 2019; Lim et al., 2019; Hu et al., 2020) and thus promote tumor metabolism.

### 3.2 The GPX4-independent pathway regulates cancer development

GCH1 is highly expressed in lung, breast and liver cancer, according to its defense against ferroptosis (Kraft et al., 2020; Liu et al., 2022). CoQH2 has also been shown to have a similar ability (Stefely and Pagliarini, 2017; Wei et al., 2021). Whether DHODH is the same remains unclear.

### 3.3 Tumor lipid metabolism and the microenvironment regulate tumor growth by ferroptosis

iPLA2β, which is overexpressed in human cancers, was recently shown to regulate ferroptosis by downregulating PUFAs (Sun et al., 2021b). ACSL3-mediated ferroptosis evasion was found in the lymphatic environment of cancer cells (Ubellacker et al., 2020). Interestingly, dysregulated cholesterol homeostasis resulting from 27 H C increases breast cancer growth by ferroptosis evasion (Liu et al., 2021), which shows that targeting the tumor microenvironment is promising in lipid metabolism because IFNγ downregulates SLC7A11 (Wang et al., 2019). When the tumor microenvironment changes, TFR1 and STEAP3 are upregulated in cancer cells, which increases iron accumulation and thus leads to ROS accumulation (Yan et al., 2021b; Ye et al., 2022).

## 4 Ferroptosis is strongly related to autophagy

**FIGURE 2:**
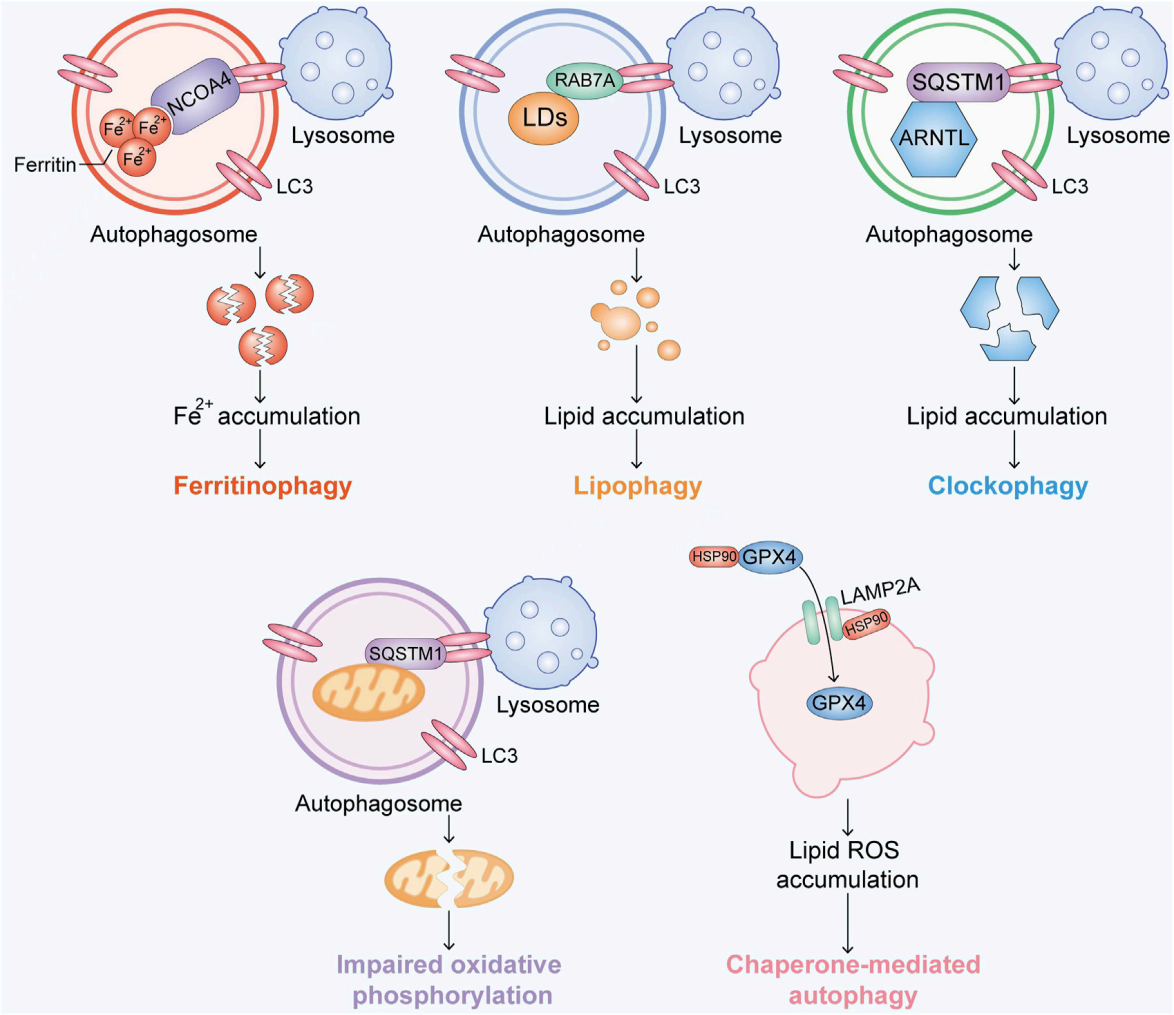
Overview of the recent research progress on autophagy-associated ferroptosis. Ferroptosis is activated by accumulating Fe, lipids or free radicals in an autophagic manner.

Researchers have recently found that the inhibition of lysosome-dependent cell death by the pharmacological blockade of cathepsin activity and vacuolar-type Hb-ATPase limits erastin-induced ferroptosis, which implies that ferroptosis is a lysosome-dependent autophagic cell death process (Gao et al., 2016; Shui et al., 2021). Subsequently, further discoveries of the link between autophagy and ferroptosis have emerged (Figure 2). The cellular redox state has a great impact on autophagy (Li et al., 2021b). Lipid peroxidation and oxidized lipids can cause MAP1-LC3 turnover as well as autophagosome formation (Hill et al., 2008; Krohne et al., 2010). Growing evidence shows that excessive autophagy or lysosome activity can lead to ferroptosis through increased iron or lipid peroxidation, which means that autophagy and ferroptosis promote each other (Kang and Tang, 2017). NCOA4 is a transcriptional coactivator of nuclear hormone receptors and undergoes gene rearrangement in cancer; this coactivator mediates ferritin degradation to lead to iron accumulation in cells and thus activate the Fenton reaction (Bellelli et al., 2014). Ferritinophagy is at the intersection of ferroptosis and autophagy, and its relationship with cancer is also expected.

### 4.1 NCOA4-associated ferritinophagy at the intersection of autophagy and ferroptosis

Ferritin is the major storage protein of iron, and its degradation is mainly carried out by lysosomes, which causes the release of iron and induces oxidative injury (Senol et al., 2008). NCOA4 is a selective cargo receptor involved in the autophagic turnover of ferritin by lysosomes (Mancias et al., 2014; Fang et al., 2021). Ferritinophagy is mediated by NCOA4, which binds FTH1, microtubule-associated protein one and LC3–PE on the developing autophagosome membrane to sequester ferritin complexes into autophagosomes (Kaur and Debnath, 2015). The flux of ferritinophagy is totally dependent on NCOA4 (Mancias et al., 2015), which is regulated by the intracellular Fe levels. The knockdown of NCOA4 or ATGs (e.g., ATG3, ATG5, ATG7, and ATG13) could suppress ferritin degradation, iron accumulation, and lipid peroxidation caused by erastin, which means that NCOA4 is possibly related to the xc-system (Hou et al., 2016). Bafilomycin A1, an inhibitor of vacuolar-type H + -ATPase (V-ATPase) in lysosomes, also inhibits ferritin degradation and ferroptosis (Zhang et al., 2018; Kong et al., 2019). These findings provide genetic evidence showing that ferroptosis is a process of selective autophagic cell death (Bellelli et al., 2016). Ferritinophagy has been shown to cause ferroptotic cell death in multiple cancer cell lines (Hou et al., 2016).

### 4.2 Non-ferritinophagy autophagy-associated ferroptosis

#### 4.2.1 RAB7A-mediated lipophagy

Lipophagy is a type of selective autophagy that causes autophagic degradation of lipid droplets (LDs) (Liu and Czaja, 2013). Importantly, lipophagy-induced LD degradation can promote lipid peroxidation in ferroptosis, and knockdown of the LD cargo receptor RAB7A or ATG5 yields the opposite result (Schroeder et al., 2015). In contrast, increased lipid storage caused by the overexpression of TPD52 reduces RSL3-induced ferroptosis (Bai et al., 2019). These findings show that the balance between lipid storage and degradation can determine the ferroptotic level.

#### 4.2.2 Clock protein ARNTL-mediated clockophagy

Clockophagy is accomplished by selective degradation of ARNTL, the core circadian clock protein, through autophagy. SQSTM1 is a freight receptor responsible for autophagic ARNTL degradation. ARNTL inhibits ferroptosis by inhibiting Egln2 transcription and thereby activating the transcription factor HIF1A, resulting in free lipid accumulation (Liu et al., 2019). The degradation of ARNTL promotes increase in the ferroptotic levels in cells.

#### 4.2.3 PINK1-mediated mitophagy

Ubiquitin is phosphorylated by the ubiquitin kinase PINK1, which activates the ubiquitin ligase Parkin, and activated Parkin builds ubiquitin chains on mitochondrial outer membrane proteins, where they recruit autophagy receptors (Yan et al., 2020). A novel model in which PINK1 produces phosphoryl ubiquitin as a mitochondrial autophagy signal and Parkin then amplifies the signal has been proven to influence the redox biology in cells and thus leads to ferroptosis (Lazarou et al., 2015; Yan et al., 2020; Chen et al., 2021).

#### 4.2.4 HSP90-mediated CMA

HSP90 has been suggested as a promising anticancer drug target due to its significance in regulating the activity and stability of many proteins in human cancers (Lanneau et al., 2007). In addition, HSP90 plays important roles in the mitochondria-dependent pathway and necroptosis (Li et al., 2015; Jacobsen et al., 2016; Zhao et al., 2016). More recently, ferroptosis has been found to be related to HSP90 (Wu et al., 2019a). Mechanistically, HSP90 regulates the stability of LAMP2A, which is an isoform of LAMP2 and a receptor for CMA (De Domenico et al., 2006; Wu et al., 2019a). LAMP2A- and HSPA8/HSC70-mediated CMA result in GPX4 degradation (Wu et al., 2019a; Lahiri et al., 2019). CDDO and tanespimycin have been shown to reduce erastin/glutamate-mediated ferroptosis in HT-22 cells. Importantly, the knockdown of HSP90 by siRNA reverses erastin/glutamate-induced ferroptosis, which means that HSP90 plays an important role in this process (Lanneau et al., 2007; Wu et al., 2019a). These results, along with the finding that HSPA5 enhances GPX4 protein stabilization, imply that HSPs play a significant role in ferroptosis.

#### 4.2.5 BECN1-mediated system xc−inhibition

The BECN1 interactome is widely known to regulate autophagy, apoptosis and many other cellular processes (Kang et al., 2011). The formation of a BECN1-SLC7A11 complex can promote the activation of ferroptosis activators such as erastin but has no influence on RSL3 or FIN56 (Kang et al., 2018; Song et al., 2018). Moreover, the phosphorylation of BECN1 enhances BECN1-SLC7A11 complex formation, inhibition of system xc-, and subsequent ferroptotic cancer cell death (Song et al., 2018). These findings imply that BECN1 is a direct inhibitor of the xc−system that induces ferroptosis.

#### 4.2.6 STAT3-mediated lysosomal dysfunction

Lysosomal dysfunction is a type of RCD (Kroemer and Jaattela, 2005). Recent studies indicate that lysosomes may play a potential role in promoting ferroptosis (Torii et al., 2016; Gao et al., 2018). Mechanistically, CTSB expression and release mediated by STAT3 are essential for ferroptosis. Importantly, integrin ITGA6-and ITGB4-mediated STAT3 activation enhances cell survival in response to erastin, which shows that different pathways can converge on STAT3 to induce ferroptosis (Gao et al., 2018). RSL3-or erastin-induced ROS generation is inhibited by lysosome inhibitors, which implies that ferroptosis is dependent on lysosomal function (Torii et al., 2016).

## 5 Ferritinophagy is regulated by many factors

### 5.1 The HERC2-FBXL5-IPR2 axis plays a main role in regulating ferritinophagy

**FIGURE 3:**
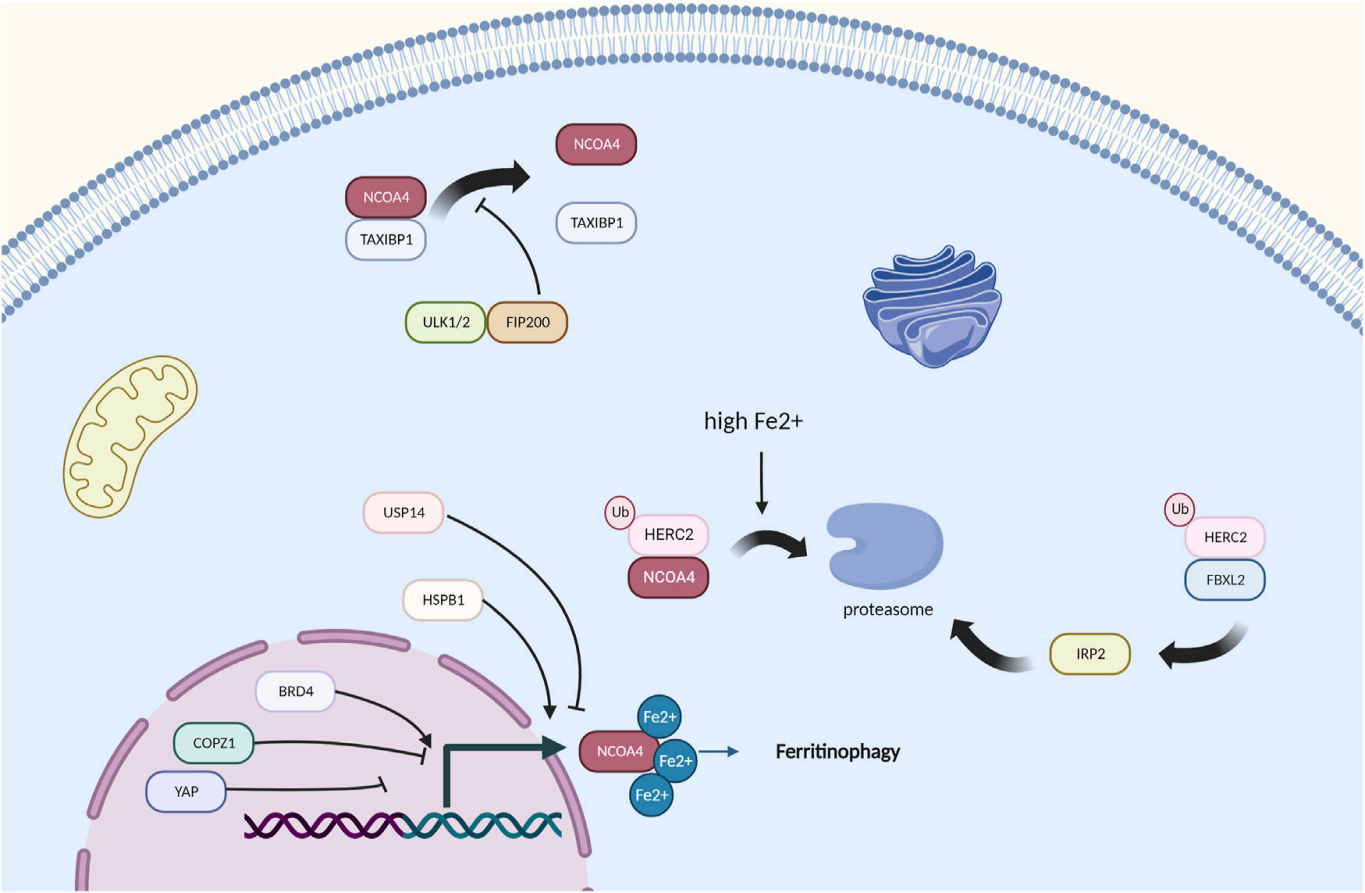
Regulators of NCOA4-mediated ferritinophagy, HERC2 and high iron pressure play a main role in the regulation of NCOA4.

NCOA4, the speed-limiting factor of ferritinophagy, has been proven to be mainly regulated by the cellular iron levels (Mancias et al., 2015). Research with the 293T, U2OS, HCT116, and K562 cell lines shows that NCOA4 binds the ubiquitin E3 ligase HERC2 and is targeted for degradation through the ubiquitin proteasome system when the cellular iron levels are high (Mancias et al., 2015) (Figure 3). This finding ensures that the NCOA4 levels are low under high iron pressure (Yao et al., 2022), which can decrease ferritinophagy and increase ferritin iron storage. In contrast, when cellular iron is low, NCOA4 and HERC2 binding is decreased, causing elevated NCOA4 levels and promoting ferritinophagic flux to restore the cellular iron levels (Ryu et al., 2018). This finding suggests that in the context of long-term NCOA4 depletion *in vivo*, the sensitivity to ferroptosis may actually be enhanced (De Domenico et al., 2006). In response to a high cellular iron level, HERC2 promotes NCOA4 turnover through the C‐terminal domain in NCOA4 and a CUL7‐homology domain in HERC2, which prevents ferritinophagy and excess liberation of iron from ferritin (Zhou et al., 2020). Additionally, HERC2 regulates the proteasomal degradation of FBXL5, which targets IRP2 for its degradation under iron overload (Moroishi et al., 2014a; Endo et al., 2017). IRP2 plays a significant role in the maintenance of cellular iron homeostasis, and the degradation of IRP2 causes a decrease in Fe2+, which thus increases the flux of ferritinophagy (Yao et al., 2021). The inhibition of HERC2-FBXL5 binding or the knockout of endogenous HERC2 by RNA interference results in the stabilization of FBXL5 and an increase in its abundance (Moroishi et al., 2014a). Thus, HERC2 ubiquitination and the FBXL5‐IRP2 axis play important roles in the NCOA4‐ferritinophagy process and control iron metabolism (Moroishi et al., 2014b), but many unanswered questions, including whether the binding of HERC2 and NCOA4 influences the binding of ferritin and NCOA4, whether other E3 ligases target NCOA4, and whether regulators of HERC2 exist, remain, and focusing on these questions will contribute to the field.

### 5.2 Ferritinophagy regulation by hypoxia

Iron is essential for Fe-S cluster formation and heme synthesis and is therefore critical for maintaining the mitochondrial respiratory chain and the citric acid cycle (Subramanian et al., 2011). However, free iron overload causes severe oxidative damage (Garcia-Bermudez et al., 2018). Under hypoxia, HIF is known to regulate iron regulatory proteins. HIF regulates not only TfR and DMT1, which are responsible for iron import but also FPN, which is the only proven iron exporter (Ma et al., 2019). FTH and FTMT have ferroxidase activity, which allows the storage of Fe3+ instead of reactive Fe2+ (Hilton et al., 2012). FTMT expression is associated with the transport of iron from the cytosol to mitochondria (Campanella et al., 2009). In addition, its expression is dependent on HIF under hypoxia. Macrophages often suffer hypoxia during inflammation and in the tumor microenvironment. Furthermore, they are widely known for their essential role in iron metabolism, although the regulation of macrophage ferritinophagy remains undefined. The overexpression of FTMT protects SH-SY5Y neuroblastoma cells from ferroptosis (Wang et al., 2016; Mendsaikhan et al., 2018). Researchers have found that the increase in FTMT in hypoxic macrophages is related to decreased NCOA4 expression caused by damaged transcriptional regulation under hypoxia, which means that ferritinophagy is directly related to hypoxia (Fuhrmann et al., 2020).

### 5.3 Identification of more regulators of NCOA4 and ferritinophagy

Wu et al. (2019b) found that curcumol reduces the expression of NCOA4 *via* Yes-associated protein (YAP), which is a key regulator in the Hippo signaling pathway and affects the formation of autophagosomes (Qi et al., 2021). COPZ1 knockdown also leads to an increase in NCOA4, resulting in ferritin degradation, an increase in intracellular ferrous iron and ultimately ferroptosis (Zhang et al., 2021). The knockdown of NCOA4 could protect cells from ART‐induced cell death, which suggests that ART is a modulator of ferritinophagy (Goodwin et al., 2017). Ferritinophagy requires ATG9A, FIP200, VPS34, and TAX1BP1, and TAX1BP1 directly binds NCOA4 and is needed for the lysosomal trafficking of ferritin under different conditions (Dowdle et al., 2014; Goodwin et al., 2017). NCOA4 is also strongly related to ubiquitination. Coimmunoprecipitation experiments have shown that TRIM11 interacts with and binds UBE2N. Mechanistically, TRIM11 promotes suppression of ferritinophagy and gemcitabine resistance through the UBE2N-TAX1BP1 pathway, and this finding demonstrates that TRIM11 is a key modulator of TAX1BP1 signaling in ferritinophagy and gemcitabine resistance, which has seen treatment effects in pancreatic ductal adenocarcinoma (Dong et al., 2019). USP14 has also been demonstrated to influence NCOA4 and has been proven to improve ischemic stroke (Hangauer et al., 2017). The absence of DNMT-1, which is significant in DNA methylation, has been proven to reduce NCOA-4-mediated ferritinophagy (Li et al., 2021c).

BRD4 and HSPB1 have been found to upregulate ferritinophagy, and BRD4 and HSPB1 are highly expressed in many cancer types (Sui et al., 2019) (Sun et al., 2022). However, how these factors actually work remains unclear. Thus, we wonder whether the targets of selective autophagy mentioned above will interact with NCOA4, and focusing on this topic will be a good research direction.

## 6 Treatments targeting ferritinophagy in cancer

Cancer cells with dedifferentiated and mesenchymal characteristics have been proven to be more susceptible to ferroptosis than noncancerous cells (Hangauer et al., 2017; Tsoi et al., 2018). Particularly in lung and breast cancer, cells are more sensitive to ferroptosis (Lei et al., 2021). Because the cancer treatments targeting ferroptosis are limited mainly to SLC7A11, treatments focusing on ferritinophagy are receiving increased attention.

Moreover, in HUVECs and EA. hy926 cells, ferritinophagy is needed for the induction of ferroptosis by ZnONPs, which are nanoparticles. Because NCOA4 knockdown alleviates ZnONP-induced cell death (Qin et al., 2021), nanoparticles provide us with an interesting and broader way of thinking.

Apart from nanoparticles, other treatments are still being investigated in the laboratory. DpdtC, a novel iron chelator, was proven to promotes ferritinophagy in HepG2 cells, and this effect has also been verified in gastric cancer (Grignano et al., 2020; Gonciarz et al., 2021). Cancer cells with dedifferentiated and mesenchymal characteristics have been proven to be more susceptible to ferroptosis than noncancerous cells (Hangauer et al., 2017; Tsoi et al., 2018). Tetrandrine citrate has also been shown to suppress breast cancer by activating NCOA4 (Yin et al., 2022). Polybrominated diphenyl ether quinone induces NCOA4-mediated ferritinophagy, and this compound may play a role in subsequent research (Yin et al., 2022). Studies in cancer cells have shown the great potential of ferritinophagic treatments in cancer. But drugs targeting the ferroptosis pathway are still in the preclinical stage, we hope to see more drugs targeting NCOA4 and HERC2 in the future.

## 7 Future therapies and challenges

Ferritinophagy has become increasingly important in ferroptosis and cancer. In this review, we describe the origin and development of ferroptosis, and the discovery of the relationships between ferroptosis and autophagy has led to the emergence of ferritinophagy. As a part of ferroptosis, this process is regulated by the cellular iron levels and is caused by iron accumulation, and as a part of autophagy, this process mediates the binding of ferritin and autophagosomes. Ferritinophagy is distinguished from other ferroptosis pathways as well as other autophagy pathways, and targeting this process has led to achievements in many types of diseases and will play a more significant role in the discovery of the mechanisms of RCD. Therefore, we note various regulators and treatments for cancer, which show the great potential of ferritinophagy in cancer treatment, and we hope to see more outcomes, which will help us better understand the mechanisms of ferritinophagy in cancer.

## Abbreviations

GPX4, Phospholipid hydroperoxide glutathione peroxidase; System xc-, Polyhydroxyalkanoic acid system protein; BAX, BCL2-associated X protein; BAK, Bcl-2 homologous antagonist; RIPK1/RIPK3, Receptor-interacting serine/threonine-protein kinase1/3; CASP1/4, Caspase-1/4; FSP1, Apoptosis-inducing factor 2; DHODH, Dihydroorotate dehydrogenase; ACSL4, Long-chain fatty acid-CoA ligase 4; GCH1, GTP cyclohydrolase 1; IPLA2β, Inositol 1,4,5-trisphosphate receptor-like protein 2β; MAP1-LC3, Microtubule-associated proteins 1A/1B light chain 3; RAB7A, Ras-related protein Rab-7a; ARNTL, Aryl hydrocarbon receptor nuclear translocator-like protein 1; HIF1A, Hypoxia-inducible factor 1-alpha; PINK1, Serine/threonine-protein kinase PINK1; PARKIN, E3 ubiquitin-protein ligase parkin; HSP90, Heat shock protein 90; BECN1, Beclin-1; HERC2, E3 ubiquitin-protein ligase HERC2; FBXL5, F-box/LRR-repeat protein 5; DMT1, Charged multivesicular body protein 2b; FTMT, Ferritin.
